# First Experience with Hypothermic Oxygenated Perfusion in Human Uteri: Feasibility and Metabolic Characterization

**DOI:** 10.3390/jcm15082820

**Published:** 2026-04-08

**Authors:** Keyue Sun, Nasim Eshraghi, Fernanda Walsh Fernandes, Sangeeta Satish, Chunbao Jiao, Fatma Selin Yildirim, Geofia Crasta, Omer F. Karakaya, Koki Takase, Hiroshi Horie, Karen S. Keslar, Dylan Isaacson, William Baldwin, Robert L. Fairchild, Koji Hashimoto, Alejandro Pita, Alvin Wee, Mariam AlHilli, Charles Miller, Mohamed Eltemamy, Tommaso Falcone, Andreas Tzakis, Elliot Richards, Andrea Schlegel

**Affiliations:** 1 Department of Inflammation and Immunity, Cleveland Clinic Research Institute, Cleveland, OH 44195, USA; eshragn@ccf.org (N.E.); fernanf5@ccf.org (F.W.F.); satishs2@ccf.org (S.S.); jiaoc@ccf.org (C.J.); yildirf@ccf.org (F.S.Y.); crastag2@ccf.org (G.C.); karakao@ccf.org (O.F.K.); takasek@ccf.org (K.T.); horieh@ccf.org (H.H.); keslark@ccf.org (K.S.K.); fairchr@ccf.org (R.L.F.); 2 Transplantation Center, Digestive Disease and Surgery Institute, Cleveland Clinic, Cleveland, OH 44195, USA; hashimk@ccf.org (K.H.); pitaa@ccf.org (A.P.); millerc8@ccf.org (C.M.); tzakisa@ccf.org (A.T.); 3 Department of Urology, Glickman Urological and Kidney Institute, Cleveland Clinic, Cleveland, OH 44195, USA; isaacsd3@ccf.org (D.I.); weea@ccf.org (A.W.); eltemam@ccf.org (M.E.); 4 Department of Obstetrics, Gynecology and Reproductive Biology, Cleveland Clinic, Cleveland, OH 44195, USA; alhillm@ccf.org (M.A.); falcont@ccf.org (T.F.); 5 Wake Forest Bapist Center for Fertility and Reproductive Surgery, Atrium Health, Winston Salem, NC 27103, USA; elliott.richards@advocatehealth.org

**Keywords:** uterus transplantation, hypothermic oxygenated perfusion, mitochondrial preservation, organ assessment, flavin mononucleotide (FMN), ischemia–reperfusion injury, donor utilization

## Abstract

**Background**: Uterus transplantation (UTx) is an emerging treatment for absolute uterine factor infertility. However, the use of deceased donors is limited, and donation after circulatory death (DCD) has not yet been utilized. Ischemic injury remains a major barrier, particularly compared with living donor procedures. Hypothermic oxygenated perfusion (HOPE), which has shown protective effects in heart, liver, and kidney transplantation, may offer similar benefits for uterine grafts. **Methods:** We report the first series applying HOPE to human uteri to improve preservation and enable metabolic injury assessment during perfusion. Six uteri (3 DBD, 3 DCD; median donor age 53 years) underwent 8 h of HOPE following procurement, while paired tissue controls were preserved using static cold storage (SCS). Perfusion was delivered using a pressure-controlled system (15 mmHg, 10 ± 1 °C, VitaSmart^®^). Perfusate and tissue samples were analyzed for mitochondrial injury, inflammation, and transcriptional responses. **Results:** HOPE maintained stable flows (70–150 mL/min), delivered high oxygen levels (pO_2_ ≈ 1000 hPa), and increased tissue ATP levels. Stratification based on perfusate flavin mononucleotide (FMN) release identified grafts with greater Complex I/II injury. HOPE was associated with lower levels of mitochondrial injury markers and inflammatory signals, preserved tissue architecture, and promoted gene expression patterns consistent with metabolic recovery compared with paired SCS tissue controls. **Conclusions:** These findings suggest that HOPE may serve as a preservation approach that enables metabolic and ischemic injury assessment and may facilitate broader use of deceased donor uteri for transplantation.

## 1. Introduction

Uterus transplantation (UTx) is the first treatment capable of restoring fertility in women with absolute uterine factor infertility [1,2]. Since the first successful live birth in 2014 in Sweden, clinical programs have expanded using both living and deceased donors and, more recently, with the introduction of robotic hysterectomy and transplantation techniques [3,4,5,6,7,8,9,10]. Despite this progress, deceased donor UTx remains limited by two main factors: the difficulty of assessing graft function before transplantation and the lack of dynamic preservation methods. To date, all uterus grafts reported with clinical transplantation have been preserved with cold storage [6]. Notably, because the uterus is the last organ removed during multiorgan procurement, it is exposed to prolonged functional donor warm ischemia (fDWIT) and extended cold ischemia times (CIT), both of which can exacerbate ischemia–reperfusion injury (IRI) and compromise graft function [11,12,13,14]. In addressing these challenges, machine perfusion has been shown to be superior for organ preservation and viability assessment in transplantation of other solid organs, such as the liver and kidneys. For example, compared to static cold storage (SCS), hypothermic oxygenated perfusion (HOPE) stabilizes mitochondrial function and prevents IRI, thereby improving post-transplant outcomes in kidney and liver transplantation, as demonstrated by randomized clinical trials [15,16,17,18]. Experimental work in uterus transplantation has been conducted primarily in sheep and porcine models. Padma et al. (2019), for instance, developed a normothermic ex vivo reperfusion model in sheep to evaluate uterine quality after cold ischemia, while porcine models have demonstrated the feasibility of hypothermic perfusion for preserving uterine tissue [19,20,21]. Despite these advances in animal studies, machine perfusion has not yet been systematically evaluated in human uteri [22].

This study evaluates the feasibility and efficacy of HOPE in the human uterus. An 8 h perfusion period was selected to mirror relevant logistics for clinical practice, allowing typical transport times plus additional viability assessment before future transplantation. We compare HOPE with conventional cold storage and assess perfusion quality, mitochondrial integrity and tissue inflammation. We hypothesize that HOPE will better maintain uterine structure and mitochondrial function compared to SCS, demonstrating its potential as an innovative, translational preservation strategy for future uterine transplantation.

## 2. Materials and Methods

### 2.1. Study Design and Donor Selection

Between June and October 2025, six human uteri underwent hypothermic oxygenated perfusion (HOPE) under institutional ethics approval with informed consent obtained from donors or next of kin in accordance with the Declaration of Helsinki. All uterus donors (median age 53 years; median BMI 29.5 kg/m^2^) had a history of successful pregnancies. Donors were classified as donation after brain death (DBD, *n* = 3) or donation after circulatory death (DCD, *n* = 3). DCD grafts experienced prolonged functional donor warm ischemia time (fDWIT).

**Figure 1:**
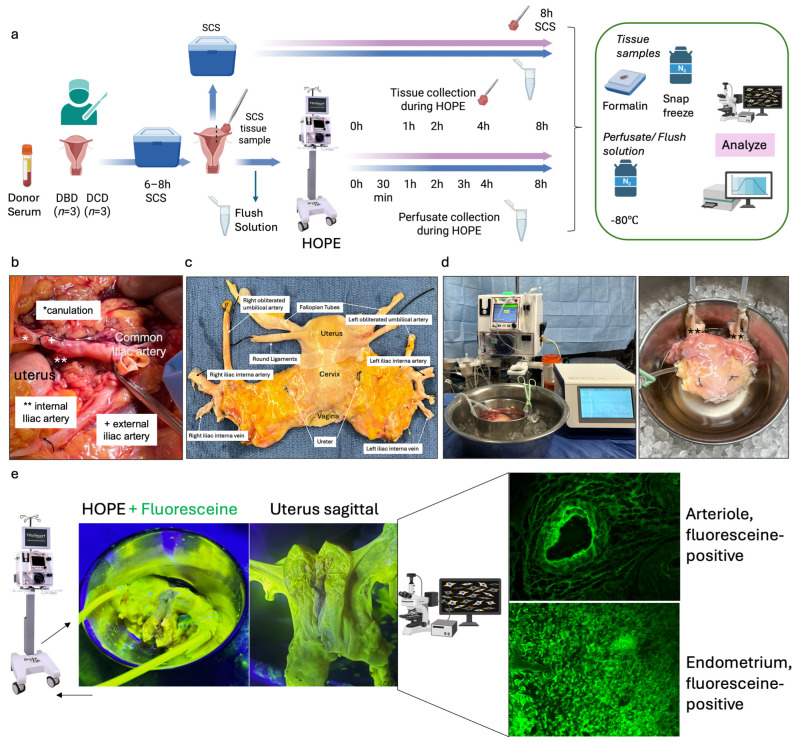
Study design and experiment flowchart. (**a**) Study design and sampling of donor serum, flush solution, and HOPE-perfusate collection at predefined timepoints. Tissue biopsies paired with cold-storage tissue controls and serial biopsies during HOPE perfusions. (**b**) In situ cold flush of the uterus via the right and left external iliac arteries in a retrograde fashion during deceased donor procurement, with clamping of the abdominal aorta to direct perfusion. (**c**) Anatomical overview of the procured uterus graft, including bilateral uterine arteries and veins in the internal iliac pedicles and a 2 cm upper vaginal cuff. (**d**) HOPE setup using the VitaSmart^®^ perfusion system (Bridge to Life, Ltd., Northbrook, IL 60062, USA), demonstrating dual cannulation of the internal iliac arteries for HOPE. (**e**) Hypothermic oxygenated perfusion quality testing using fluorescein. Rapid and homogenous tissue perfusion and oxygenation with HOPE was confirmed by macroscopic and microscopic imaging. Vascular structure and cells in all uterus tissue layer are perfused entirely.

Functional donor warm ischemia time was defined as the interval between systolic blood pressure <50 mmHg and initiation of cold perfusion. While other solid organs concomitantly procured from the same donors had a median fDWIT of 24 min, the median fDWIT for the uterus was 52 min (Table 1). All uteri were flushed in situ with histidine–tryptophan–ketoglutarate (HTK) and cold stored for a median of 8.5 h prior to 8 h of HOPE. Paired SCS controls represent tissues collected immediately from each uterus (*n* = 6) before HOPE and after additional SCS in preservation solution at 4 °C for 8 h instead of HOPE (Figure 1a).

### 2.2. Uterus Procurement Procedure

For DBD donors, a cold HTK flush was initiated within 3–5 min via the common or external iliac arteries, followed by en bloc procurement of the uterus, uterine vessels with internal iliac pedicles, and a 2 cm vaginal cuff [13,22,23].

For DCD donors, laparotomy was performed after circulatory death and 5 min stand-off period. Routine cold donor flush was initiated through the aorta. Of note, effective DCD donor uterus flush and procurement is currently performed only after other solid organs are flushed and procured (median uterus fDWIT 52 min). Subsequent procurement steps were identical to DBD donor procurement. The common iliac arteries or the external iliac arteries were cannulated, and the lower abdomen/femoral axis was flushed, followed by en bloc uterus procurement as described for DBDs above. All grafts received a 1 L cold HTK back-table flush before storage and transport on ice. HOPE was then initiated after transport on ice and was combined with paired SCS sampling (Figure 1b,c).

Hypothermic oxygenated perfusion (HOPE) of human uterus

Before initiating HOPE, each organ was flushed with 300 mL of Belzer MPS^®^ (Bridge to Life, Ltd., Northbrook, IL 60062, USA). All six uteri were perfused using the VitaSmart^®^ device (Bridge to Life, Ltd., Northbrook, IL 60062, USA), at 15 mmHg and 8–12 °C with 700 mL Belzer MPS^®^ and continuous oxygenation. Flow, pressure, temperature, O_2_ (PyroScience GmbH, 52072 Aachen, Germany), and CO_2_ (13CORLab, ArgosMED GmbH, 76185 Karlsruhe, Germany) were continuously monitored. Perfusion was maintained for 8 h (Figure 1). Commercially available Belzer Machine perfusion solution (MPS) was used for HOPE across all experiments.

Static Cold Storage Controls

The paired uterine tissue biopsies were collected from the right anterior and posterior walls at the top-corpus level before starting HOPE. Each biopsy was about 10 × 10 mm and included all uterine layers. Samples were sharply excised with a scalpel for consistency. Paired uterine tissue was preserved in Belzer MPS^®^ at 4 °C for 8 h. During perfusion, the biopsy site was monitored and no significant leakage or impairment of perfusion flow was observed (Figure 1a).

Sample Collection and Biochemical Analyses

Donor serum, flush effluent, perfusate, and cold-storage solution were collected at predefined timepoints (Figure 1a). Tissue biopsies were snap-frozen or formalin-fixed. Flavin mononucleotide (FMN) and nicotinamide adenine dinucleotide (NADH) were quantified by fluorescence spectroscopy [24,25]. Succinate, mitochondrial Complex I and II, HMGB-1, and 8-OHdG were measured in donor serum and perfusate using ELISA (AssayGenie, BA0149, Dublin 2, D02 VY42, Ireland; Mybiosource Inc. San Diego, CA 92195-3308, USA, MBS93108630, MBS9336905; HMGB-1, 30164033, IBL America, Minneapolis, MN 55432, USA; AB285254, abcam, Waltham, MA 02453, USA;). Tissue ATP content was assessed with a luminescence-based assay [26]. Gene expression was analyzed using the NanoString Human Metabolic and Inflammation Panels, with differential expression defined as |log_2_FC| ≥ 1 and *p* < 0.05. NanoString analysis was performed on paired tissue samples from a subset of two uteri collected at baseline, after 8 h of HOPE, and after 8 h of static cold storage (SCS). Differential expression analysis was performed using paired comparisons between preservation conditions within each donor. Targeted metabolomic profiling was performed on snap-frozen uterine tissue collected at baseline, after 8 h of HOPE, and paired tissue after 8 h of SCS. Metabolites were extracted using a methanol–water protocol and analyzed via LC-MS-based targeted metabolomics (pyruvate, lactate, malate, succinate, citrate/isocitrate, ADP, NAD, hypoxanthine, xanthine). Peak intensities were normalized to tissue wet weight to account for biopsy-to-biopsy variability. Data were visualized as a normalized heatmap to compare metabolic signatures across preservation conditions.

Histology and Immunohistochemistry

Formalin-fixed tissues were paraffin-embedded and stained for hematoxylin-eosin (H&E) to assess tissue structure, necrosis and structure, and Galectin-3 for inflammation and fibrosis. Complex I (anti-NDUFS1; ABN302, abcam, Waltham, MA 02453, USA) immunohistochemistry was performed on uterus tissues to assess mitochondrial injury.

Statistical Analysis

All measurements were performed in duplicate and reported as medians with interquartile ranges (IQRs). Group comparisons used the Mann–Whitney U test. Exploratory correlation analysis was performed using Pearson’s correlation coefficient. A *p*-value < 0.05 was considered statistically significant. Because this study included only six uteri, all statistical analyses were considered exploratory. Analyses were performed with GraphPad Prism 10.4.1 (Boston, MA 02110, USA).

## 3. Results

### 3.1. HOPE of the Human Uterus

Six human uteri (DBD: *n* = 3; DCD: *n* = 3) were included between June and October 2025. The median donor age was 53 years. All uteri underwent HOPE for 8 h. Differences in recorded graft weight were primarily related to variable removal of surrounding parametrial adipose tissue during back-table preparation rather than intrinsic differences in uterine size. The perfusion was performed at 10 ± 1 °C with a median pressure of 15 mmHg and flow rates of 70–150 mL/min. Continuous inflow oxygenation remained around 1000 hPa throughout. All grafts were successfully perfused for the entire 8 h without technical issues. To assess the perfusion quality during HOPE, fluorescein was added to the perfusate (concentration: 0.286 mg/mL). Microscopic assessment showed a highly fluorescein-positive uterine tissue, especially the vascular structure and endometrium (Figure 1e).

### 3.2. Mitochondrial and Inflammatory Responses During HOPE

**Figure 2:**
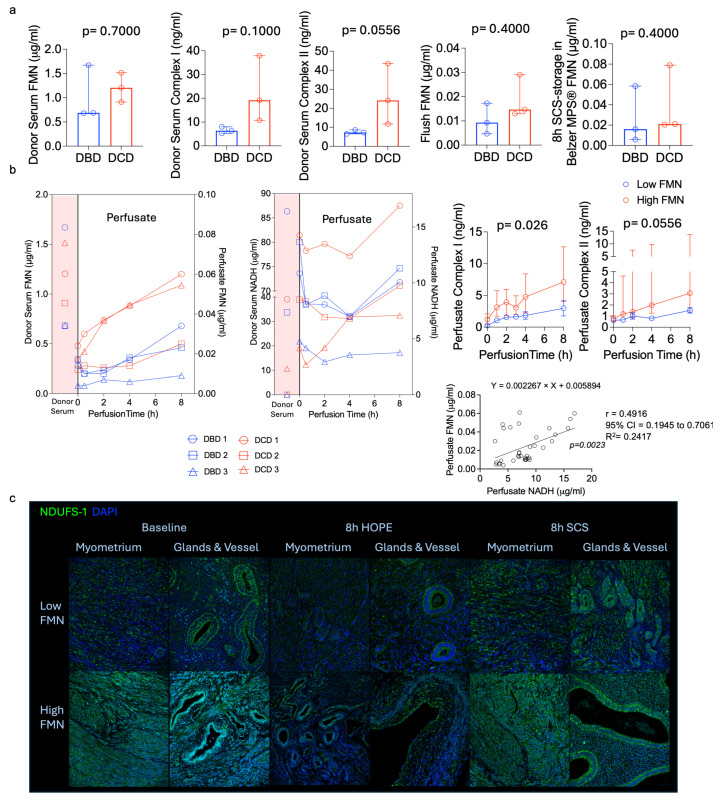
Mitochondrial injury in organ donor and during HOPE of human uterus. (**a**) Donor serum samples collected from DCD donors showed higher FMN levels and other markers of mitochondrial injury, reflecting more pronounced injury compared to DBD donor uteri. The flush solution obtained after a median of 8.5 h of cold storage and the cold storage solution of paired SCS uterine tissue demonstrated the same pattern. (**b**) DCD grafts showed higher baseline FMN levels compared with DBD grafts, which correlated with greater FMN/NADH release over 8 h HOPE, with an increase in perfusate FMN after 4 h. Donor serum FMN correlated directly with graft FMN levels during HOPE and reflected overall graft injury. Using a perfusate FMN cutoff of ≤0.025 µg/mL, grafts were stratified into high-FMN (*n* = 4) and low-FMN (*n* = 2) groups. Perfusate NADH correlated with perfusate FMN (*p* = 0.0023), although FMN appeared to perform better as an injury marker. FMN-based stratification showed clear correlation with other mitochondrial injury markers, including Complex I, and Complex II. These correlations suggest that FMN may reflect mitochondrial injury. (**c**) Representative immunohistochemistry for Complex I (anti-NDUFS1) comparing grafts with low versus high FMN release. High (risk)-FMN uteri exhibit stronger Complex I positivity consistent with greater mitochondrial injury, whereas low (risk)-FMN grafts show minimal NDUFS1 positivity. HOPE-treated uterus shows reduced Complex I activation compared with baseline and static cold storage.

**Figure 3:**
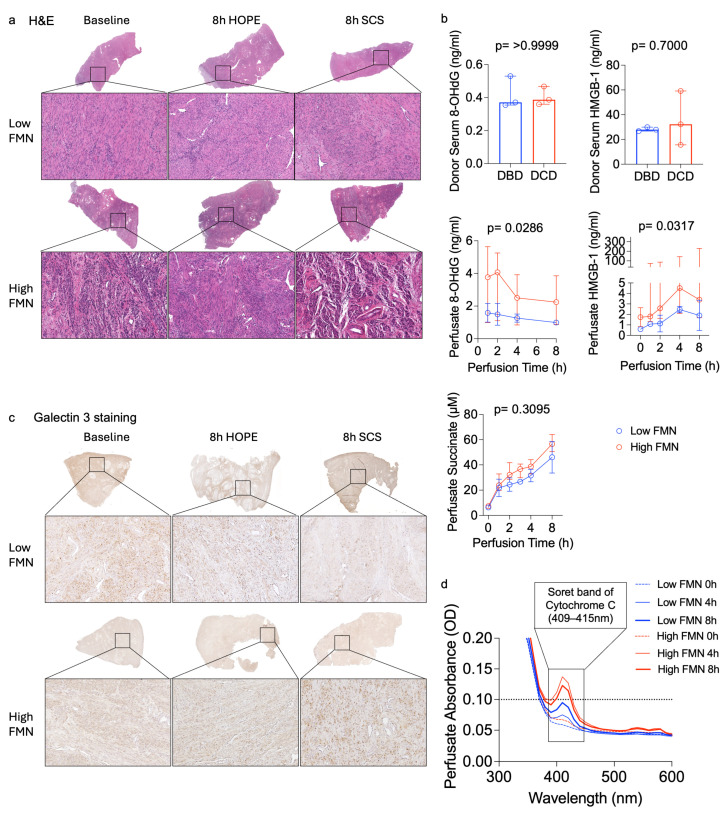
Inflammatory response and histological changes during HOPE of human uterus in correlation with perfusate FMN release. (**a**) In the H.E. staining, the high-FMN group shows overall more oedema and disrupted cellular architecture compared to the low-FMN group. However, HOPE treatment appears to restore cellular structure and the condition of smooth muscle cells. (**b**) FMN-based stratification showed similar patterns across other inflammatory markers including succinate, HMGB-1 and 8-OHdG. (**c**) Galectin-3 staining also showed greater positivity in the high-FMN group. (**d**) Absorbance spectroscopy revealed changes in the development of the Soret band during HOPE. The high (risk)-FMN group demonstrated increased cytochrome-c-associated spectral peaks and a Soret band at 409–415 nm, suggesting increased mitochondrial respiratory chain stress.

**Figure 4:**
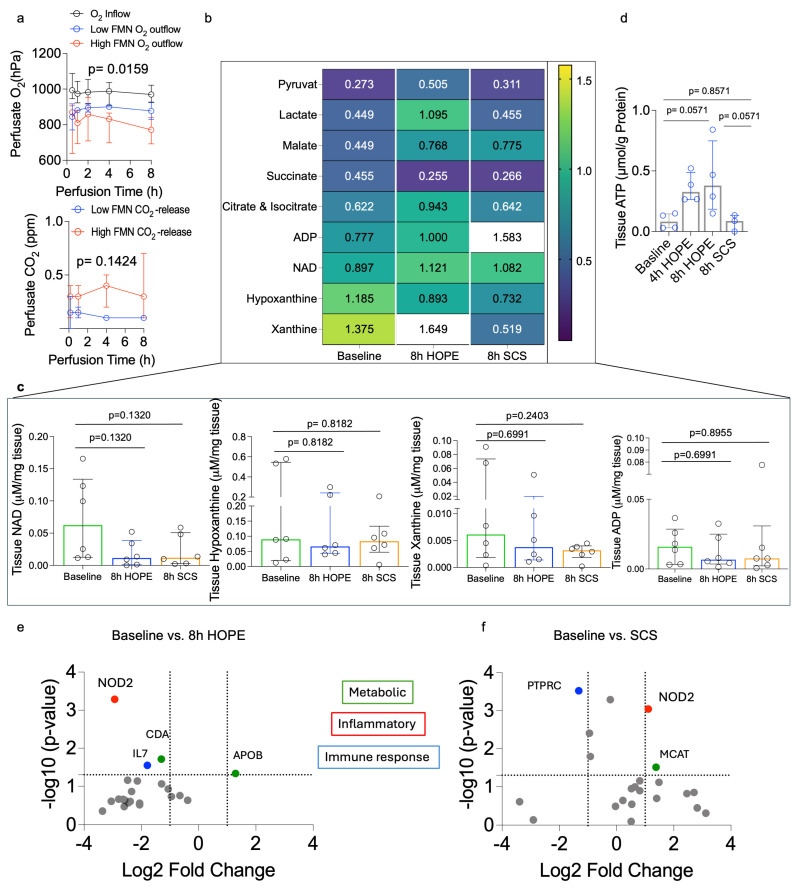
Metabolic status and gene expression in uterus tissue. (**a**) Oxygen consumption and CO2 release of uterus during HOPE. High-FMN grafts demonstrated both increased O_2_ consumption and higher CO_2_ production, consistent with TCA-cycle overrun and elevated metabolic stress. (**b**) Heatmap of metabolite concentrations measured at baseline, 8 h HOPE, and SCS. Values represent relative metabolite abundances for key intermediates of glycolysis, the TCA cycle, adenine nucleotide metabolism, and purine degradation. Warmer colors represent higher concentrations, cooler colors lower concentrations. Notably, HOPE was associated with increased levels of pyruvate, lactate, citrate/isocitrate, and with higher NAD levels compared to baseline. In contrast, SCS generally exhibits lower metabolic turnover and lower levels of purine degradation products. (**c**) Concentrations of NAD, ADP, hypoxanthine, and xanthine in uterus tissue before HOPE, after 8 h of HOPE, and after 8 h of SCS (median, IQR). (**d**) Following 4 h and 8 h HOPE treatment, the graft demonstrates a higher ATP recharge in the tissues compared to both baseline and SCS groups. (**e**,**f**) NanoString gene-expression profiling of human uteri preserved by HOPE or SCS. Volcano plots compare (**e**) baseline versus 8 h HOPE and (**f**) baseline versus 8 h SCS. The *x*-axis shows log_2_ fold change, and the *y*-axis shows −log_10_ (*p*-value). Dotted lines indicate thresholds for significance (*p* < 0.05; |log_2_FC| ≥ 1). Genes are color-coded by functional category: inflammatory (red), metabolic (green), and immune response (blue).

DCD uteri, which were exposed to a substantially longer fDWIT (median 52 min) compared to the other procured organs from the same donors (median fDWIT 24 min), showed higher perfusate FMN and NADH levels compared with DBD organs (Figure 2). Despite these differences, HOPE perfusion was associated with metabolic recovery indicators in both donor types. Analysis of donor serum and uterine flush samples from DCD donors showed consistently elevated levels of mitochondrial injury markers (FMN, Complex I, Complex II). Higher levels of HMGB-1 and 8-OHdG indicating inflammation and oxidative DNA damage further underline the increased baseline injury seen in DCD donors (Figure 2 and Figure 3). HOPE was associated with increasing ATP levels during perfusion, suggesting preservation of mitochondrial metabolic activity (Figure 4c).

During HOPE, FMN remained higher in DCD grafts. However, variability was observed across all organ donors (Figure 2b). In contrast, the cold-storage flush solution from paired SCS controls showed minimal differences between the DBD and DCD groups (Figure 2a). For exploratory analysis, a perfusate FMN cutoff of ≤0.025 µg/mL was used to stratify uteri into high (risk)-FMN (*n* = 4) and low (risk)-FMN (*n* = 2) groups. In this exploratory analysis, ELISA-based markers including Complex I, Complex II, succinate, HMGB-1, and 8-OHdG showed concordant trends with FMN stratification, consistent with an association between higher FMN release and greater mitochondrial injury (Figure 2 and Figure 3). Additionally, absorbance spectroscopy demonstrated higher cytochrome-c-associated spectral peaks and the Soret band around 409–415 nm in the high-FMN group, further indicating increased mitochondrial respiratory chain stress (Figure 3d). O_2_ consumption correlated positively with CO_2_ production and with perfusate FMN release, suggesting that organs with a higher Complex I injury also had higher O_2_ requirements due to greater oxidative stress (Figure 4a). Although some of these differences did not reach statistical significance due to the small sample size, consistent trends were observed across the measured markers.

### 3.3. Histologic and Immunohistochemical Findings

Histologic evaluation demonstrated that HOPE-preserved uteri maintained intact endometrial and myometrial architecture with minimal oedema. In contrast, SCS organs showed cellular swelling, and myometrial oedema (Figure 3a). Immunohistochemistry corroborated these observations. Complex I staining, an indicator of respiratory chain activation and injury under IRI-stress, was lower in HOPE-treated tissue compared to uteri with prolonged SCS. Baseline uterus tissue with high FMN release showed stronger Complex I positivity, whereas grafts with low FMN release showed minimal positivity. After 8 h of HOPE, the Complex I positivity was lower relative to SCS, indicating reduced mitochondrial stress during HOPE (Figure 2c). Galectin-3 staining was consistently higher in SCS samples, reflecting increased inflammation (Figure 3c). Overall, HOPE-treated uteri displayed lower mitochondrial injury across all markers, consistent with improved structural and cellular preservation.

### 3.4. Gene Expression Responses (NanoString)

NanoString analysis showed that HOPE was associated with only small shifts in gene expression. Inflammatory and immune-related genes, including *NOD2*, *IL7*, and *PTPRC*, were downregulated. At the same time, HOPE was associated with modest upregulation of selected metabolic genes, including *APOB*. In contrast, SCS was associated with broader transcriptional changes compared with baseline, characterized by increased expression of immune and inflammatory genes such as *PTPRC* and *NOD2*, along with higher levels of metabolic and stress-related genes including *MCAT*. Overall, HOPE was associated with lower inflammatory gene expression than SCS (Figure 4e,f).

### 3.5. Mass Spectrometry

HOPE promotes a metabolic profile consistent with mitochondrial protection, including slower and more efficient succinate metabolism, preserved TCA-cycle intermediates, and lower purine degradation products compared with SCS. In contrast, SCS showed metabolic signatures of greater ischemic stress, including elevated hypoxanthine/xanthine and less favorable TCA-cycle and nucleotide metabolites (Figure 4b,d).

### 3.6. Comparison of HOPE and Static Cold Storage (SCS)

Across all biochemical and histological endpoints, HOPE was associated with improved metabolic and structural preservation compared with SCS. ATP levels increased during perfusion, reflecting sustained mitochondrial respiration during HOPE. The uterus tissue architecture was better preserved after HOPE treatment with reduced oedema, and lower Complex I and II activation, whereas SCS grafts showed structural deterioration and stronger inflammatory signals and expression of mitochondrial injury. Collectively, HOPE was associated with biochemical and structural features suggestive of improved preservation compared with SCS tissue controls.

## 4. Discussion

Uterus transplantation (UTx) has developed into a promising treatment option for women with absolute uterine factor infertility. However, broader clinical implementation remains limited by the low utilization of deceased donor (DD) organs, particularly those from donation after circulatory death (DCD) donors. To our knowledge, this study represents the first report describing hypothermic oxygenated perfusion (HOPE) in human deceased donor uteri and describes the associated metabolic and mitochondrial responses during perfusion. To date, DCD donors have not been used in uterus transplantation due to concerns about prolonged ischemic injury and the lack of reliable preservation and graft assessment strategies. A major challenge in deceased donor UTx is prolonged ischemic exposure during multi-organ procurement. Because the uterus is often procured after life-saving organs, it experiences prolonged warm ischemia. Preservation still relies primarily on static cold storage (SCS), and validated strategies for graft preservation and objective assessment remain lacking. Improving preservation and evaluation approaches may play an important role in expanding the use of deceased donor uteri.

In this study, HOPE perfusion was technically feasible in all grafts. It was associated with a more favorable metabolic profile compared with SCS. Similar findings have been reported in other solid organs. In those cases, HOPE was associated with biochemical patterns consistent with preserved mitochondrial metabolism. During perfusion, ATP levels increased while markers of oxidative stress and tissue injury remained relatively stable. These observations indicate that uterine tissue stays metabolically active under hypothermic oxygenated conditions. This observation is consistent with the prolonged ischemic exposure associated with uterus procurement, particularly in DCD donors. Ischemic stress can damage mitochondria and lead to cellular injury. This may lead to the release of metabolites, including FMN, succinate, and other components of the respiratory chain. Despite this baseline injury, all grafts in our series maintained metabolic activity during HOPE. Targeted metabolomic analysis supported these observations. It demonstrated limited succinate accumulation, preserved TCA-cycle intermediates, and reduced purine degradation products such as hypoxanthine and xanthine compared with SCS. These metabolic patterns are consistent with findings reported in HOPE studies of liver and kidney grafts [16,26,27,28]. Perfusion was initiated at 15 mmHg with a target flow of 150 mL/min; transient adjustments were required in selected cases to establish adequate arterial inflow, after which perfusion was maintained at the target pressure. These variations reflect practical aspects of machine perfusion in a real-world setting. Importantly, flow and pressure remained stable throughout perfusion, and these variations did not affect overall perfusion quality or the study results.

Machine perfusion in uterus transplantation remains relatively unexplored, with only a few experimental studies published to date. Most existing normothermic and hypothermic perfusion approaches have concentrated on physiological observations rather than standardized preservation protocols, viability assessments, or links to post-transplant outcomes [21,29].

Another observation from this study was that the donor category alone did not predict uterine graft quality. Both DBD and DCD groups included grafts with variable metabolic profiles. While DCD grafts showed higher baseline injury, donor category alone did not fully explain the variability in metabolic profiles observed across grafts. This suggests that donor category alone may not adequately reflect graft condition. It highlights the potential value of individualized, perfusion-based assessment of graft status. In the DCD group, the prolonged functional donor warm ischemia time (fDWIT), with a median of 52 min, was largely related to delayed uterine flushing. This delay aimed to avoid interfering with the procurement of life-saving organs. Although HOPE appeared to stabilize the metabolic parameters in these grafts, prolonged warm ischemia remains a concern. These observations emphasize the importance of refining DCD procurement logistics. Refinement could include introducing earlier vascular access or parallel organ retrieval strategies.

Our observations are consistent with experience from liver, kidney, and heart transplantation. In those organs, HOPE has been shown to reduce ischemia-reperfusion injury and improve early graft function, especially in extended-criteria and DCD organs [15,16,29,30,31,32]. HOPE has also been associated with reduced release of damage-associated molecular patterns (DAMPs), decreased endothelial activation, and modulation of inflammatory responses. Although immune mechanisms were not directly examined in the present study, these effects may be relevant for uterus transplantation. UTx recipients are typically young women who require immunosuppression if the graft remains in place. Preservation strategies that reduce early ischemic injury may help limit inflammatory activation. They may also support future efforts to minimize exposure to immunosuppression [33,34,35].

Flavin mononucleotide (FMN) has recently been proposed as a marker of mitochondrial injury during liver perfusion [36]. Similar patterns were observed in uterine grafts in this study. FMN levels correlated with NADH and other components of the respiratory chain. When grafts were stratified by perfusate FMN levels, trends consistent with mitochondrial stress emerged. Together, these observations indicate that FMN may represent a candidate marker of mitochondrial injury during uterine perfusion. At present, however, these findings remain exploratory. No uterus-specific FMN thresholds have been established. The clinical significance of these observations cannot be determined without post-transplant outcome data. While HOPE may provide a platform for metabolic monitoring, functional validation through reperfusion models and clinical studies will be required.

Beyond preservation, HOPE may help address practical barriers to deceased donor UTx, such as prolonged preservation times, limited operative planning windows, and the lack of objective graft assessment tools. Future studies should further explore how HOPE could be integrated into clinical uterus transplantation workflows, including its potential role in extending preservation time and enabling organ assessment. HOPE may represent a strategy to expand the donor pool, improve preservation logistics and viability assessment enabling the use of extended-criteria DBD and DCD uterus grafts.

Several limitations should be acknowledged. The number of grafts was small, and therefore all analyses should be interpreted as exploratory. In addition, no reperfusion model or post-transplant functional data were available, preventing correlation of perfusion biomarkers with functional uterine outcomes. Hormonal responsiveness, contractile activity, and other physiological parameters relevant to uterine viability were not evaluated. Furthermore, the comparison with static cold storage relied on paired tissue biopsies rather than whole-organ preservation controls, which may not fully reflect the physiological conditions of conventional graft storage. Only one preservation solution and perfusion protocol were tested, and optimal parameters for uterine machine perfusion remain to be further defined. In addition, the impact of donor vascular disease and other risk factors on uterus quality should be assessed to potentially broaden the number of available deceased donors. Future studies should include larger cohorts and correlate perfusion biomarkers, including FMN, with clinically relevant outcomes such as graft survival, menstruation, and pregnancy. Accordingly, the observations of this study should be interpreted as hypothesis-generating and require confirmation in larger experimental and clinical studies.

In summary, HOPE was associated with improved preservation by supporting mitochondrial metabolism and reducing biochemical markers of ischemic injury during perfusion. These findings provide initial human evidence that hypothermic oxygenated perfusion may serve as a preservation and metabolic assessment platform for deceased donor uterus grafts. Future studies incorporating reperfusion models, functional validation, and larger cohorts will be essential to determine whether machine perfusion can facilitate safer utilization of deceased donor uteri and support broader clinical application of uterus transplantation.

## Figures and Tables

**Table 1:** Uterus donor and graft characteristics—overview of all uteri included in the study (*n* = 6). Representing images show each uterus after procurement and during hypothermic oxygenated perfusion (HOPE). Donor demographics (age, BMI, pregnancy history), donor category (DBD, DCD), warm ischemia time, hysterectomy duration, cold storage time and graft weights are listed.

Uterus Donors	DBD 1	DBD 2	DBD 3	DCD 1	DCD 2	DCD 3
Uterus before HOPE	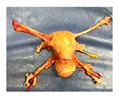	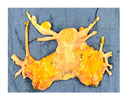	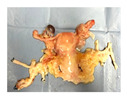	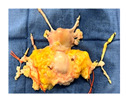	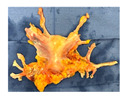	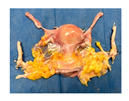
Uterus during HOPE	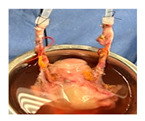	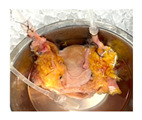	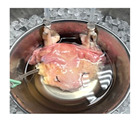	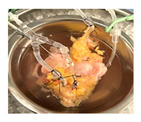	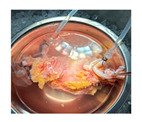	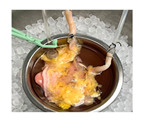
Risk factors	–	–	–	–	–	–
Donor Age	38	55	54	53	40	59
Gravidity and parity	G2/P2	G2/P2	G1/P1	G2/P2	G3/P3	G2/P2
Donor BMI (kg/m^2^)	33.3	36.3	25.3	18.2	25.6	35.4
Total DWIT (min)	–	–	–	24	24	27
Functional DWIT (min)	–	–	–	24	22	24
Asystolic DWIT	–	–	–	20	18	3
Total uterus WIT (min)	–	–	–	84	74	82
Functional uterus WIT (min) *	–	–	–	60	44	52
Asystolic uterus WIT	–	–	–	84	68	58
Hysterectomy time (min) **	107	137	123	110	112	108
Static cold storage (min)	397	540	540	420	598	470
Graft weight (g)	70	230	230	180	170	210
Gynecologic findings	N/A	Two right paratubal cysts	Suggestive of ovarian endometrioma adjacent to the right fallopian tube	N/A	N/A	N/A

*: time from cross clamp to uterus flush; **: time from cross clamp to hysterectomy.

## Data Availability

The data used to support the findings of this study are included and available within the article. Additional data are available from the corresponding author upon reasonable request.

## Abbreviations

The following abbreviations are used in this manuscript:

8-OHdG	8-hydroxy-2′-deoxyguanosine
ADP	adenosine diphosphate
APOB	apolipoprotein B
ATP	adenosine triphosphate
BMI	body mass index
CDA	cytidine deaminase
CIT	cold ischemia time
CO_2_	carbon dioxide
Complex I	mitochondrial NADH:ubiquinone oxidoreductase
Complex II	mitochondrial succinate dehydrogenase
CSF3R	colony-stimulating factor 3 receptor (G-CSF receptor)
DAMPs	damage-associated molecular patterns
DBD	donation after brain death
DCD	donation after circulatory death
ELISA	enzyme-linked immunosorbent assay
FMN	flavin mononucleotide
FPR2	formyl peptide receptor 2
fDWIT	functional donor warm ischemia time
H&E	hematoxylin and eosin
HMGB1	high mobility group box 1
HOPE	hypothermic oxygenated perfusion
HTK	histidine–tryptophan–ketoglutarate
ICAM4	intercellular adhesion molecule 4
IHC	immunohistochemistry
IL7	interleukin 7
IQR	interquartile range
IRI	ischemia–reperfusion injury
KYNU	kynureninase
LC–MS	liquid chromatography–mass spectrometry
LTB	lymphotoxin β
MCAT	malonyl-CoA–acyl carrier protein transacylase (mitochondrial)
MPS^®^	Machine Preservation Solution (Belzer MPS^®^)
NAD	nicotinamide adenine dinucleotide (oxidized form)
NADH	nicotinamide adenine dinucleotide (reduced form)
NanoString	NanoString nCounter^®^ gene expression profiling system
NDUFS1	NADH:ubiquinone oxidoreductase core subunit S1 (Complex I subunit)
NOD2	nucleotide-binding oligomerization domain–containing protein 2
O_2_	oxygen
PCA	principal component analysis
PTPRC	protein tyrosine phosphatase receptor type C (CD45)
S100A12	S100 calcium-binding protein A12
SCS	static cold storage
SDH	succinate dehydrogenase (Complex II)
TCA	tricarboxylic acid cycle
UTx	uterus transplantation
UW	University of Wisconsin solution
